# Rapid and Highly Efficient Method for Scarless Mutagenesis within the *Salmonella enterica* Chromosome

**DOI:** 10.1371/journal.pone.0015763

**Published:** 2011-01-14

**Authors:** Kathrin Blank, Michael Hensel, Roman G. Gerlach

**Affiliations:** 1 Junior Research Group 3, Robert Koch Institute, Wernigerode Branch, Wernigerode, Germany; 2 Division of Microbiology, School of Biology/Chemistry, University of Osnabrück, Osnabrück, Germany; New England Biolabs, United States of America

## Abstract

Direct manipulation of bacterial chromosomes by recombination-based techniques has become increasingly important for both cognitive and applied research. Here we demonstrate, for the first time, the combination of the Red recombinase system with I-*Sce*I endonuclease-based selection of successful recombinants after electroporation with short synthetic olignucleotides. We show the generation of scarless gene knockouts as well as site-directed mutagenesis using the *Salmonella* virulence-associated two component signaling system PhoPQ. The presented approach is very versatile for generating in-frame deletions, point mutations or insertions within bacterial chromosomes.

## Introduction

The generation of markerless deletions and mutations within a bacterial genome is of increasing importance for both cognitive and applied research. Techniques based on λ Red-mediated recombination were previously shown to be very versatile for generating gene deletions in *Escherichia coli* [1], *Salmonella enterica* [2], *Pseudomonas aeruginosa* [3] as well as *Shigella* spp. [4] and *Yersinia enterocolitica* [5] or *Y. pestis* [6]. Beyond the manipulation of bacterial genomes, the technology has recently emerged as a tool for manipulating bacterial artificial chromosomes (BACs) in various applications.

Homologous recombination implies technologies of gene targeting, an introduction process of recombinant DNA into specific sites mediated by endogenous recombination machinery, and recombineering which refers to the engineering of recombinant DNA [7]. The Red recombinase system of phage λ consists of Redα, a 5′ to 3′ exonuclease, Redβ, a single strand annealing protein and Recγ, which inhibits the host exonuclease RecBCD [7]. Homologous recombination catalyzed by λ Red is remarkably efficient using linear DNA with homology extensions of only 36 to 40 bp in length [1], [8]. With this technique, a chromosomal sequence of varying length is replaced by a selectable antibiotic resistance marker [1]. In an optional second step, this antibiotic resistance cassette can be removed, thus generating markerless deletions. The system developed by Datsenko and Wanner used expression of Flp recombinase which acts on corresponding Flp recombinase target (FRT) sites to excise the resistance marker from the genome leaving a 81 to 85 bp “scar” sequence [1]. Although there is limited homology of the scar sequence itself, it could provide a site for homologous recombination itself which might lead to problems and unwanted results in further manipulations. In addition, this scar might interfere with gene function when modifying operon structures or intragenic regions. Recently, we presented a method able to generate scarless deletions which is based on two successive λ Red recombination steps [9]. In the first step, we integrated a tetracycline resistance cassette in the target region, and in the second step, short synthetic oligonucleotides are used to remove this antibiotic marker. The use of oligonucleotides for λ Red-mediated recombination omits the requirement of constructing mutant alleles as targeting constructs (TCs) for recombination [9]. Integrating a tetracycline resistance cassette in the first step enabled us to select for successful recombinants after the second recombination using growth suppression of Tet^r^ clones on Bochner-Maloy plates [9], [10]. Bochner-Maloy plates contain inactivated chlortetracycline, the lipophilic chelator fusaric acid and Zn^2+^ ions which potentiate the effects of fusaric acid [11]. The exact mechanism how growth of tetracycline-resistant clones is inhibited on Bochner-Maloy plates is still unknown. Since this selection procedure is not very stringent, it requires exact timing of incubation steps and results sometimes in high background making purification of positive clones difficult. Thus, here we present a modification of this method allowing much easier and efficient selection of recombinants with the help of I-*Sce*I-induced double-strand breaks (DSB). I-*Sce*I is an intron-encoded endonuclease of the yeast *Saccharomyces cerevisiae* having an unusual long recognition sequence of 18 bp [12]. Because of the statistical absence of natural I-*Sce*I recognition sites within bacterial genomes, the enzyme was previously used as a tool for genetic engineering [13], [14], [15].

To establish the new approach we targeted the two-component regulatory system PhoPQ of *Salmonella enterica* serovar Typhimurium (*S*. Typhimurium). PhoPQ was shown to influence the expression of a vast set of virulence-associated genes in *Salmonella* [16]. PhoQ is a histidine sensor kinase responding to different environmental stimuli such as low pH, low phosphate or presence of antimicrobial peptides and is thought to detect the host environment [17]. A mutation within the *phoPQ* locus called *pho-24* or *phoP* ^c^ results in constitutive activation of the PhoP response regulator [18]. *pho-24* is a single nucleotide mutation leading to a Thr to Ile exchange at position 48 (T48I) within the periplasmic domain of PhoQ [19]. Constitutive activation as well as inactivation of the PhoP regulon attenuates *Salmonella* virulence in macrophages and mice [18]. One of the PhoP-activated genes (*pag*) encodes for the nonspecific acid phosphatase PhoN [20]. Phosphatase activity of *S*. Typhimurium is linked to PhoN expression levels and can easily be determined by the use of chromogenic substrates [21]. Introduction of T48I in or deletion of *phoQ* has high impact on *phoN* expression [18]; which we used as a model to facilitate screening and phenotypic characterization of recombinants.

We successfully constructed a system consisting of the template vector pWRG100 and pWRG99, a temperature-sensitive plasmid for independent inducible expression of the λ Red recombinase and I-*Sce*I endonuclease. In a first step, primers with 40 bp homology extensions were used to amplify the chloramphenicol resistance cassette and an I-*Sce*I recognition site from pWRG100. This construct was integrated within *phoQ* via recombination. In a second step, 80mer dsDNAs, derived from oligonucleotides, were used for (I) deletion of *phoQ* and (II) site-directed mutagenesis of *phoQ*. Integrity of both constructs was verified by sequencing and phenotypic characterization. The method presented here provides easy and convenient access to modify bacterial chromosomes, as well as cosmids and BACs, shown to be amenable for the Red recombinase system. Manipulations possible include in-frame, scarless deletions, site-directed mutagenesis, but also generation of C- and N-terminal fusions.

## Results

**Figure 1:**
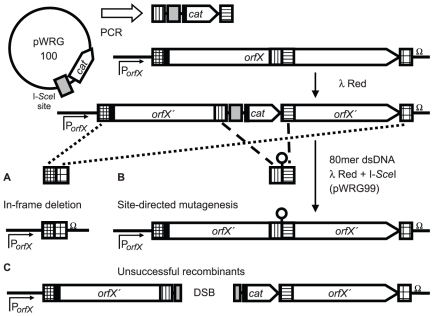
Rationale of the recombineering method. A chloramphenicol resistance cassette (*cat*) is amplified together with an I-*Sce*I recognition site (grey bars) from plasmid pWRG100 by PCR using primers with 40 bp homology extensions (vertical and horizontal hatching). After integration within a target gene “*orfX*” via λ Red-mediated recombination, the mutant can be used (A) to generate an in-frame deletion: A 80mer dsDNA is designed to comprise sequences homologous to flanking regions of *orfX* (cross-hatched). (B) Site-directed mutagenesis of the region adjacent to the I-*Sce*I recognition site: is done with the help of a synthetic 80mer mutant dsDNA fragment harboring the same homologous regions used for integration of the resistance cassette (vertical and horizontal hatching). In a second λ Red-mediated recombination step the 80mer dsDNA is integrated within the genome. Use of plasmid pWRG99 at this point allows for sequential expression of I-*Sce*I after recombination which leads to effective killing of unsuccessful recombinants by induction of DSBs (C).

An overview about the principle of our technique is given in Fig. 1: In the first step, a chloramphenicol resistance cassette (*cat*) together with an I-*Sce*I recognition site is inserted using λ Red recombinase-mediated recombination into the target locus (*orfX*). After confirming proper insertion of the resistance cassette by colony PCR, 80mer DNA fragments derived from oligonucleotides are electroporated in the mutant strain expressing the λ Red recombinase system. The 80mer DNA fragments can contain regions homologous to flanking regions of *orfX* leading to in-frame deletions (Fig. 1A) or mutant alleles spanning the resistance cassette for site-directed mutagenesis (Fig. 1B). By using suitable oligonucleotides, codons can be inserted within an ORF in the same way (not shown). Selection of successful recombinants is mediated by sequential expression of I-*Sce*I endonuclease after recombination. If no recombination occurred, the unique I-*Sce*I restriction site within the genome is still present and a DSB is induced after induction of I-*Sce*I expression. Thus, these clones can not replicate leading to efficient killing of bacteria (Fig. 1C).

**Figure 2:**
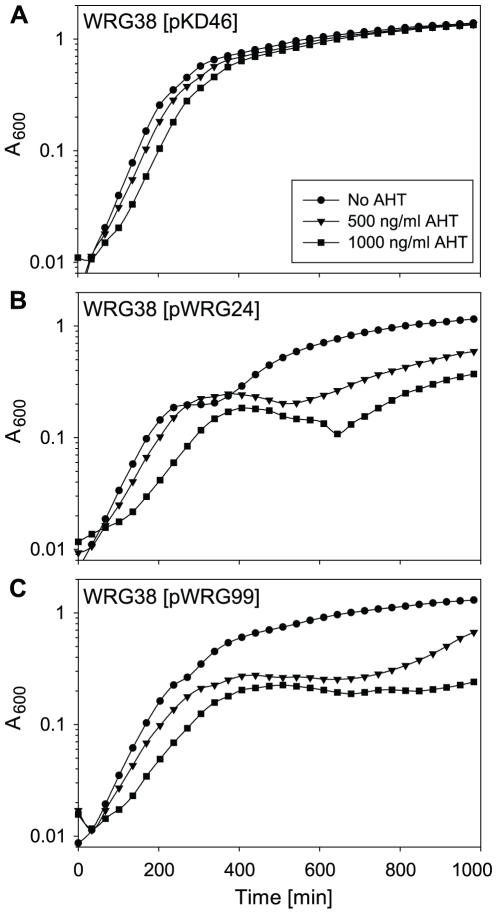
Growth of WRG38 (*phoQ*150::*cat* I-*Sce*I) harboring both variants of pKD46-I-*Sce*I or pKD46 without and with addition of AHT. Depicted are growth curves of strain WRG38 harboring pKD46 (A), pKD46 with tetracycline-inducible I-*Sce*I cassette in orientation one (5'->3': *tetR*-I-*Sce*I, pWRG24) (B) or in orientation two (5'->3': I-*Sce*I-*tetR*, pWRG99) (C). Overnight cultures were adjusted to OD_600_ = 0.01 in LB with 50 µg/ml carbenicillin (circles), further supplemented with 500 ng/ml (triangles) or 1000 ng/ml (squares) AHT. Growth curves were recorded over 16 hours using a Bioscreen C device at 30°C. Without AHT, *S*. Typhimurium [pWRG24] (B), shows a more drastic growth defect whereas the strain with pWRG99 (C) is only slightly attenuated in growth compared to WRG38 [pKD46] (A). Addition of AHT has a slight inhibitory effect on growth as shown by pKD46 controls (A). Both, pWRG24 (B) and pWRG99 (C) show a significant growth inhibition upon addition of AHT due to expression of I-*Sce*I and induction of DSB. At an AHT concentration of 500 ng/ml pWRG99 showed higher inhibitory effect compared to pWRG24. 1000 ng/ml AHT nearly abolished growth of WRG38 [pWRG99] (C).

The template vector for polymerase chain reaction (PCR) pKD3 contains a selectable antibiotic resistance gene (*cat*, Cm^r^) flanked by FRT sites [1]. We added an I-*Sce*I recognition site to this plasmid which was then designated pWRG100. To achieve an independently controllable expression of I-*Sce*I and λ Red recombinase system, we modified the Red recombinase expression plasmid pKD46 [1]. pKD46 is a low copy, temperature-sensitive plasmid and expression of the λ Red system (Redαβγ) can be induced by addition of 10 mM L-arabinose [1]. The pKD46 derivatives pWRG24 (*tetR*-I-*Sce*I) and pWRG99 (I-*Sce*I-*tetR*) harbor the endonuclease I-*Sce*I under control of a tetracycline-inducible promoter. Both orientations of pKD46-I-*Sce*I were compared with parental pKD46 in their ability to mediate Red recombination using primers PhoQ-Mut-for and PhoQ-Mut-rev and template pWRG100 to generate TCs. The homology extensions added to the 5′ ends of the primers target the construct to be inserted at codon 50 of *phoQ* which is in close proximity to codon 48 (*pho-24*). In case of site-directed mutagenesis, the antibiotic resistance cassette should be inserted as close as possible to the site to be mutated. Following the method described previously [9], both plasmid orientations had recombination efficiencies comparable to pKD46 (not shown). However, we found bacteria harboring pWRG24 were attenuated in growth (Fig. 2B). The resulting strain WRG38 featured an I-*Sce*I recognition site and a Cm^r^ cassette inserted at position 150 of *phoQ*. Strain WRG38 was cured from plasmids pWRG24 or pWRG99 by incubation at 37°C. Alternatively, *phoQ*150::*cat*/I-*Sce*I mutation was transferred by means of P22 HT105/1 *int-201* phage transduction to a fresh WT background.

To test whether I-*Sce*I function can be induced with addition of anhydrotetracycline (AHT), we analyzed the growth of WRG38 harboring pWRG24 or pWRG99 after induction in LB. As a control, WRG38 [pKD46] was included and showed only mild dose-dependent growth defects upon addition of AHT (Fig. 2A). In contrast to this, both strains having I-*Sce*I under control of the tetracycline-inducible promoter showed a growth inhibition with 500 and 1000 ng/ml AHT (Fig. 2B and 2C). We observed that strain WRG38 with pWRG99 was more attenuated compared to WRG38 [pWRG24]. The growth of WRG38 harboring pWRG99 was nearly abolished by the addition of 1000 ng/ml AHT (Fig. 2C). For the selection procedure on plates, we rather used 500 ng/ml AHT because the growth inhibition with 1000 ng/ml AHT was too strong resulting in very low numbers of clones (not shown). Because of its greater efficiency and the growth defect of pWRG24-harboring strains (Fig. 2B), functionally equivalent pWRG99 was used in all further experiments.

**Figure 3:**
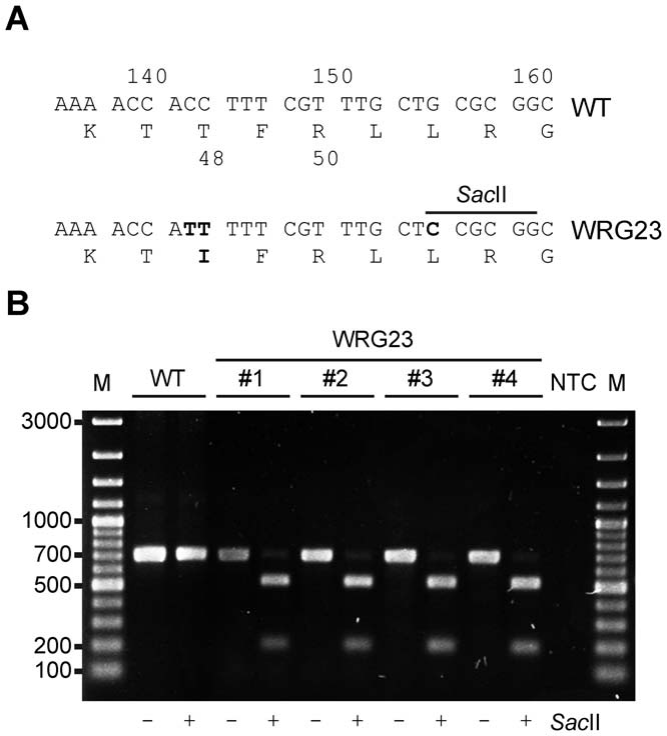
Introduction of the T48I allele within chromosomal *phoQ* of *S*. Typhimurium. (A) Sequence of the target region within *phoQ* in WT and mutated WRG23 strain. Nucleotide positions are indicated above and codon positions below sequence. Changed nucleotides and amino acids are in bold. Mutations C143T and C144T introduced the T48I (*pho-24*) mutation and G156C generated a novel *Sac*II restriction site without changing the amino acid sequence. (B) PCR products of 693 bp for a *phoPQ* fragment flanking the mutation were amplified of representative deep blue colonies (#1–4) and WT. The products were subjected to restriction analyses with *Sac*II as indicated. The expected fragments of 179 bp and 514 bp could be detected in all clones tested. M = DNA marker; NTC = non template control.

For scarless recombineering, 80mer oligonucleotides were designed including 40 bp flanking regions of *phoQ* or the T48I allele. In the latter case, a silent mutation generating a novel *Sac*II restriction site was identified *in silico* within codon 52 of *phoQ* using WatCut (http://watcut.uwaterloo.ca/) and the oligo sequences were modified accordingly [9] (Table 1, Fig. 3A). If no phenotypic screening system for recombinants is available, a novel restriction site can be used for initial colony screening. The *Sac*II site represents a second mutation in close proximity to T48I and both should be introduced simultaneously. For maximum recombination efficiency, resistance cassette was inserted between both mutation sites at codon 50 and 80mer oligonucleotides were designed to harbor the exchanged nucleotides within their central parts. Solutions of 100 pmol/µl of oligonucleotides PhoQ-Mut-Scarless-for and PhoQ-Mut-Scarless-rev were mixed equally and annealed as described previously [9]. Resulting double-stranded (ds) 80 bp DNA was phosphorylated with T4 polynucleotide kinase (Fermentas, St. Leon-Rot, Germany) following manufacturers instructions. Alternatively, 5' phosphorylation can be opted for primer synthesis. Using phosphorylated TCs, we observed a greatly enhanced efficiency of the method (data not shown). It was recently shown that phosphorylation of the 5' end of one strand of the TCs improves λ Red-mediated recombination [7]. After retransfer of pWRG99 in WRG38, electrocompetent cells were generated with λ Red recombinase induced by addition of 10 mM L-(+)-arabinose. 500 to 1000 ng of phosphorylated dsDNA were used for transformation as described previously [9]. After incubation at 30°C for 1 hr, 100 µl of 10^−1^ to 10^−4^ dilutions were plated onto LB agar plates containing 500 ng/ml AHT and 50 µg/ml carbenicillin. Introduction of T48I mutation in the resulting strain WRG23 was visualized by supplementary addition of 40 µg 5-Bromo-4-chloro-3-indolyl-phosphate toluidine salt (BCIP) per plate and deep blue colonies, indicating overexpression of PhoN, were selected. Successful recombinants or suppressors formed distinct larger colonies and background of smaller colonies could be reduced with dilution. Usually 60 to 80% of the larger colonies were deep blue. If AHT was omitted from the plates or pKD46 was used instead of pWRG99, very low amounts of deep blue colonies with no differences in colony size could be observed. Results of three independent experiments are summarized in Table 2. After overnight incubation at 30°C, single colonies were purified and successful recombination was checked by absence of antibiotic resistance, colony PCR and restriction analysis. For screening, a 693 bp fragment was amplified from genomic DNA of deep blue colonies using primers PhoQ-DelCheck-for and PhoP-DelCheck-rev in a PCR. The PCR fragments were subsequently subjected to restriction analysis using *Sac*II. Analysis of four clones is shown in Fig. 3 (B) where the newly introduced *Sac*II site within the mutant strain WRG23 led to two fragments of 179 and 514 bp, respectively. All the clones of WRG23 identified by colony PCR could be further confirmed by sequencing (not shown). Finally, pWRG99 was cured by restreaking colonies and incubation at 37°C.

**Table 1:** Oligonucleotides used in this study.

Oligonucleotide	Sequence (5'→3'), restriction sites underlined, I-*Sce*I site italic
Cloning primers	
NcoI-tetR-for	CGTCCATGGGGAAAAAGGTTATGCTGCTTTTA
NcoI-I-SceI-rev	CGTCCATGGGACCAATTCGGGTCGACTTATTA
pKD-for	GTGTAGGCTGGAGCTGCTTC
pKD3-I-SceI-XbaI-rev	TTCTCTAGA *CTATATTACCCTGTTATCCCTAGCGTAACT*AAGTATAGGAACTTCGGCGC
XhoI-PhoPQ-for	GACCTCGAGAAGAGTTGACCCGTGGCAAG
PhoPQ-HindIII-rev	GCGAAGCTTGATGCTTAACGAGATGCGTG
Deletion primers	pWRG100 homologous ends italic
PhoQ-Mut-for	GGTCGGCTATAGCGTAAGTTTTGATAAAACCACCTTTCGT*CGCCTTACGCCCCGCCCTGC*
PhoQ-Mut-rev	TGGCGAGGGTATAAAACAGGTTGCTTTCGCCGCGCAGCAA*CTAGACTATATTACCCTGTT*
Control primers	
PhoQ-DelCheck-for	AAGAGGTAATGGCGCGTATG
PhoQ-DelCheck-rev	GAGCGGATCGATAAAGTTGC
PhoP-DelCheck-rev	CATTCCGGTTGAATGCTTTT
Oligonucleotides for recombination	changed nucleotides bold, new *Sac*II site underlined
PhoQ-Del-Scarless-for	TACCACCGTACGCGGACAAGGATATCTTTTTGAATTGCGCTAAACATTTCTGTGCGTTCTTCCACGCATCTCGTTAAGCA
PhoQ-Del-Scarless-rev	TGCTTAACGAGATGCGTGGAAGAACGCACAGAAATGTTTAGCGCAATTCAAAAAGATATCCTTGTCCGCGTACGGTGGTA
PhoQ-Mut-Scarless-for	GCGCTGGTCGGCTATAGCGTAAGTTTTGATAAAACCA**TT**TTTCGTTTGCT**C** CGCGGCGAAAGCAACCTGTTTTATACCCT
PhoQ-Mut-Scarless-rev	AGGGTATAAAACAGGTTGCTTTCGCCGCG**G**AGCAAACGAAA**AA**TGGTTTTATCAAAACTTACGCTATAGCCGACCAGCGC

**Table 2:** Quantification of colonies with constitutive active PhoP phenotype after recombination.

WRG38+Plasmid	500 ng/ml AHT	Number of large colonies at 10^−1^ dilution	% large colonies with deep blue phenotype
pKD46	+	0	<0.23%±0.06% (of total colonies)
pWRG99	−	0	<1.03%±1.31% (of total colonies)
pWRG99	+	78±46	71.46%±10.82%

Annealing and subsequent phosphorylation of 80mer oligonucleotides PhoQ-Del-Scarless-for and PhoQ-Del-Scarless-rev resulted in a TC with sequences homolog to regions adjacent to *phoQ*. We used strain WRG38 and the method described above to generate mutants deficient for *phoQ*. Big colonies obtained after I-*Sce*I-induced selection of successful recombinants were screened by colony PCR and we found a similar fraction of clones being positive comparable to the site-directed mutagenesis (not shown). The genotype of the resulting strain WRG6 was confirmed by sequencing. This demonstrates the usefulness of the approach for markerless in-frame deletions.

**Figure 4:**
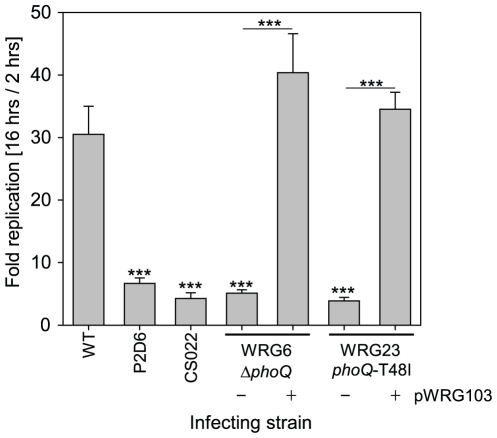
Quantification of intracellular survival within RAW264.7 macrophage-like cells using gentamicin protection assay. The intracellular replication rates of the mutant strains WRG6 (Δ*phoQ*) and WRG23 (*phoQ*-T48I) were compared to *S*. Typhimurium WT and the previously characterized mutants P2D6 (*ssaV*::mTn*5*) and CS022 (*pho-24*). After infection with a MOI of 5 non-internalized bacteria were killed by gentamicin treatment and the intracellular bacteria were quantified 2 h and 16 h after infection by plating of cell lysates. Intracellular replication rates are expressed by the quotient of 16 h colony forming units (CFU) and the 2 h CFU. One representative of three independent experiments done in triplicates is shown. Statistical analysis by Student's *t*-test was done by comparison with the WT or by comparing individual strains as depicted: *** *P*<0.001.

For further phenotypic characterization of the obtained mutants, we used RAW264.7 mouse macrophage-like cells in an infection model and gentamicin protection assays as described [22] to quantify intracellular survival. Using a multiplicity of infection (MOI) of 5, constitutive active PhoQ (WRG23) as well as a *phoQ* deletion (WRG6) were attenuated comparable to the *ssaV*-deficient strain P2D6 which cannot translocate SPI-2 effectors [23] or the previously described T48I*-*harboring strain CS022 (Fig. 4) [18]. For complementation WRG6 and WRG23 were transformed with the low-copy plasmid pWRG103 containing P*_phoP_*-*phoPQ*. With introduction of pWRG103 intracellular replication rates of WRG6 as well as WRG23 could be restored to WT levels (Fig. 4).

## Discussion

With the method described here, we present a fast and convenient way to introduce markerless deletions as well as nucleotide exchanges into the *Salmonella* genome. We employ a combination of λ Red recombination and I-*Sce*I-induced DSB for counter-selection. Introducing chromosomal DSBs will induce the microbial host DSB repair response. This repair mechanism recombines regions of sequence homology flanking the DSB and should be considered when manipulating repetitive sequences. A method published by Yu *et al*. utilizes this mechanism to introduce markerless deletions in a single step in *E. coli* [15]. This mechanism was also exploited to manipulate BACs [24]. As a consequence, we used primer binding sites different from those published by Datsenko and Wanner [1] to amplify the TCs from pWRG100. Otherwise, recombination between flanking FRT sites instead of λ Red-mediated recombination of the TC would occur frequently (data not shown).

We demonstrated an easy and convenient way to perform site-directed mutagenesis within the *Salmonella* genome. The introduction of the T48I mutation within PhoQ resulted in constitutive activation of PhoP and corresponding phenotypes as previously described [18]. In this mutant strain, WRG23, three nucleotides were exchanged simultaneously with recombination of an 80mer dsDNA. Two of these exchanges (C143T and C144T) changed codon 48 from ACC to ATT resulting in the T48I mutation (Fig. 3). The third exchange, G156C, generated a novel *Sac*II restriction site used for screening. Such a silent exchange is recommended if no phenotypic screen of resulting mutants is available. If mutants can be screened phenotypically, our method might be also applied to do random mutagenesis of defined regions using 80mers with randomized cores flanked by homologous regions. The use of synthetic oligonucleotides for recombination circumvents any cloning steps and provides maximum flexibility for introducing mutations and screening markers. Previously published methods for *E. coli* [25] and *Salmonella* Enteritidis [13] use overlapping extension PCRs and/or cloning to generate TCs and rely on co-electroporation of the TC together with an I-*Sce*I expressing plasmid. Our method allows the independent expression of the λ Red system and I-*Sce*I from the temperature-sensitive plasmid pWRG99 (Fig. 1).

## Materials and Methods

### Cloning

All oligonucleotides used are listed in Table 1. Template plasmid pWRG100 was generated using pKD3 [1] as template in a PCR with primers pKD-for and pKD3-I-SceI-XbaI-rev. The resulting fragment harboring the chloramphenicol resistance gene and an I-*Sce*I recognition site was cloned into pKD3 backbone via *Xba*I. The orientation of the resistance gene according to original pKD3 and its integrity was verified by restriction analysis and sequencing (not shown). Functionality of I-*Sce*I recognition site was proven by digestion with recombinant I-*Sce*I (Fermentas, St. Leon-Rot, Germany) (data not shown). The λ Red- and I-*Sce*I-expressing plasmids pWRG24 and pWRG99 are derivatives of pKD46 [1]. The gene for I-*Sce*I under control of a tetracycline-inducible promoter (P*_tetA_*/*tetR*) was amplified by PCR from plasmid pST98-AS [14] using primers NcoI-tetR-for and NcoI-I-SceI-rev and cloned in pKD46. Clones of both orientations, pWRG24 (*tetR*-I-*Sce*I) and pWRG99 (I-*Sce*I-*tetR*), were isolated and approved by sequencing. For complementation of strains WRG6 and WRG23 a low-copy plasmid harboring *phoPQ* under its natural promoter was generated. A fragment containing P*_phoP_*-*phoPQ* was amplified by PCR from wild-type (WT) genomic DNA using primers XhoI-PhoPQ-for and PhoPQ-HindIII-rev. The PCR product was cloned into low-copy pWSK29 [26] leading to pWRG103. All constructs were verified by restriction analysis and DNA sequencing and introduced in competent cells by electroporation (MicroPulser, Bio-Rad, Munich, Germany).

### Bacterial strains, plasmids, media, chemicals and oligonucleotides

Bacterial strains used are listed in Table 3 and plasmids in Table 4. Strains were selected using media containing 50 µg/ml carbenicillin (Roth, Mannheim, Germany), 34 µg/ml chloramphenicol (Roth) or 50 µg/ml kanamycin (Roth), where appropriate. AHT and L-arabinose were purchased from Sigma-Aldrich (Schnelldorf, Germany). BCIP was from Biomol (Hamburg, Germany). Oligonucleotides used for recombination and cloning were all HPLC-purified and purchased from Thermo Scientific (Ulm, Germany).

**Table 3:** Bacterial strains used in this study.

Strain	Relevant characteristic(s)	Source or Reference
*S. enterica* serovar Typhimurium strains		
NCTC 12023	Wild type, Nal^S^, isogenic to ATCC 14028	NCTC, Colindale, UK
CS022	*phoQ*-T48I (*pho-24*), changed nucleotide in *phoQ*: C143T	[18]
P2D6	*ssaV*::mTn*5*, Km^r^	[23]
WRG6	Δ*phoQ*	This study
WRG23	*phoQ*-T48I (*pho-24*), *Sac*II; changed nucleotides in *phoQ*: C143T, C144T, G156C	This study
WRG38	*phoQ*150::*cat* I-*Sce*I, Cm^r^	This study

**Table 4:** Plasmids used in this study.

Plasmid	Relevant characteristic(s)	Source or Reference
pKD46	λ Red-expression under control of arabinose-inducible promoter, temperature-sensitive, Amp^r^	[1]
pKD3	Red template plasmid, suicide vector (ori R6K), Amp^r^, Cm^r^	[1]
pST98-AS	Low-copy-number vector, I-*Sce*I endonuclease under control of tetracycline-inducible promoter (P*_tetA_*), Amp^r^	[14]
pWSK29	Low-copy-number vector, Amp^r^	[26]
pWRG24	pKD46 with I-*Sce*I endonuclease under control of tetracycline-inducible promoter (P*_tetA_*), temperature-sensitive, orientation 1 (5'->3': *tetR*-I-*Sce*I), Amp^r^	This study
pWRG99	pKD46 with I-*Sce*I endonuclease under control of tetracycline-inducible promoter (P*_tetA_*), temperature-sensitive, orientation 2 (5'->3': I-*Sce*I-*tetR*), Amp^r^	This study
pWRG100	pKD3 with I-*Sce*I recognition site, Cm^r^	This study
pWRG103	P*_phoP_-phoPQ* in pWSK29, Amp^r^	This study

### Growth curves

Overnight cultures of strains were adjusted to OD_600_ = 0.01 in LB supplemented with antibiotics where appropriate using a BioPhotometer plus (Eppendorf, Hamburg, Germany). A total volume of 400 µl of the inoculum was added to one well of a 100 well honeycomb plate (Growth Curves Ltd., Helsinki, Finland) and strains were assayed in triplicates. Growth curves were recorded over 16 hours using a Bioscreen C device (Growth Curves Ltd.) at 30°C with linear constant shaking intensity set to 80 and measuring absorbance at 600 nm (A_600_) every 5 minutes.

### Cell culture and infection

RAW264.7 mouse macrophage-like cells (LGC Standards, Wesel, Germany) were grown in DMEM (PAA, Pasching, Austria) supplemented with 2 mM GlutaMax (Invitrogen, Karlsruhe, Germany) under humidified atmosphere with 5% CO_2_. Gentamicin protection assays were essentially carried out as described previously [22] using a multiplicity of infection (MOI) of 5. Intracellular bacteria were quantified after 2 and 16 hrs by plating on LB plates using a spiral plater (WASP, Don Whitley Scientific, Shipley, UK) in combination with automated colony counting (aCOLyte, Synbiosis, USA).

A detailed step-by-step protocol of the presented method can be found in Supporting Materials S1 provided online.

## Supporting Information

Supporting Materials S1 **Rapid and highly efficient method for scarless mutagenesis within the** ***Salmonella enterica*** **chromosome.**
(DOC)
