# Supplementary material for: Rapid and Highly Efficient Method for Scarless Mutagenesis within the Salmonella enterica Chromosome
Source: PLoS One. 2011 Jan 14;6(1):e15763. doi: 10.1371/journal.pone.0015763 (PMC3021506; doi:10.1371/journal.pone.0015763)
Supplement: Supporting Materials S1 — Rapid and highly efficient method for scarless mutagenesis within the Salmonella enterica chromosome. (DOC) [file pone.0015763.s001.doc]

**Supplementary Materials S1**

**Rapid and highly efficient method for scarless mutagenesis within the *Salmonella enterica* chromosome**

**Authors:** Kathrin Blank^1^, Michael Hensel^2^ and Roman G. Gerlach^1^

^1^Junior Research Group 3, Robert Koch-Institute, Wernigerode Branch, D-38855 Wernigerode, Germany

^2^Division of Microbiology, School of Biology/Chemistry, University Osnabrück, D-49076 Osnabrück, Germany

**Detailed step-by-step protocol for scarless recombineering in *S*. Typhimurium**

**Approximate time needed for completion of the method: 10-12 days**

**Primer design for insertion of I-*Sce*I with resistance cassette (Cm^r^):**

- Parts homologous to pWRG100 (I-*Sce*I-*cat*) 5'→3'

Forward: CGCCTTACGCCCCGCCCTGC

Reverse: CTAGACTATATTACCCTGTT

- Add 40 bp of site-specific sequence for recombination
- Order HPLC purified primers

**PCR to amplify I-*Sce*I with resistance cassette (Cm^r^):**

- Use polymerase with proof reading activity (e.g. Fermentas HiFi, Eurogentec AccuStar, Finnzymes Phusion) according to manufacturer's instructions
- Add template plasmid pWRG100 ~2 ng/50 µl reaction mix
- Optimize for high annealing temperature
- 30 cycles 100 µl (2 x 50 µl)
  - Expected fragment: 1000 bp (with 40 bp homology extensions)
- Optional: Do *Dpn*I digestion to remove traces of pWRG100 template:

This step might be omitted, because pWRG100 is a derivative of suicide plasmid pKD3. Therefore, contaminations with template plasmid do not occur at high frequency.

- Purify and concentrate PCR products with suitable PCR cleanup kits and columns (e.g. Qiagen PCR Purification Kit, Macherey Nagel NucleoExtract II), elute in 30 µl elution buffer
- Optional: DNA precipitation for higher DNA concentrations

**Insertion of I-*Sce*I with resistance cassette (Cm^r^) in *S*. Typhimurium**

- Prepare electrocompetent cells of *S*. Typhimurium WT as described before [1]
- Transform pKD46 and use immediately
- Prepare electrocompetent cells (use glucose-free LB, ultra-clean glassware or plastic ware, ultra-clean water e.g. Milli-Q) and induce λ Red system with 10 mM arabinose
- Transform 200-500 ng of PCR product (e.g. use Bio-Rad Micropulser with”EC2” setting)
- Select transformants on LB + Cm^34^ plates at 37°C

**Verification of mutants and curing of pKD46**

- Colony PCR using suitable check primers (e.g. C1 or C2 [2] with site-specific primers)
- Curing of pKD46 either by restreaking and incubation at 37°C over night

Or

- Transduction of the mutation via P22 [3] in a fresh WT background
- Test clones for carbenicillin sensitivity

**Design and annealing and phosphorylation of 80mer dsDNA targeting constructs**

- Design 80 bp primer containing:
  - (I) 40 bp flanking a deletion site
  - (II) A mutated allele within its central part

It is recommended to introduce a novel restriction site within the mutant allele without changing amino acid sequence to facilitate screening. The online tool “WatCut” (http://watcut.uwaterloo.ca/) provides such a function for primer design.

- Order HPLC-purified primer plus reverse complement primer with 5' phosphorylation and dissolve to 100 pmol/µl
- Mix equal amounts (at least 10 µl) primer and reverse complement primer in a safe-lock tube and anneal both to obtain dsDNA:
  - Heat water bath to 95°C
  - Incubate primer mix for 15 minutes
  - Switch off water bath and let cool down over night
- If no phosphorylated primers were ordered, phosphorylate dsDNA targeting construct (according to Fermentas):
  - Mix X µl of annealed oligos (~20 pmol 5` termini)
  - Add 2 µl 10x reaction buffer A
  - Add 2 µl 10mM ATP
  - Add 2 µl T4 polynucleotide kinase (PNK, Fermentas)
  - Add water to 20 µl
  - Incubate at 37°C for 30 min
- Optional: DNA precipitation for higher DNA concentrations

**Generation of mutants of *S*. Typhimurium with selection using I-*Sce*I-induced double-strand breaks**

- Use freshly prepared *S*. Typhimurium mutant with I-*Sce*I recognition site and chloramphenicol resistance harboring pWRG99
- Prepare electrocompetent cells (use glucose-free LB, ultra-clean glassware or plastic ware, ultra-clean water e.g. Milli-Q) and induce λ Red system with 10 mM arabinose
- Transform 500-1000 ng of annealed oligos (dsDNA)
- Select transformants on LB + Cb^50^ plates containing 500 ng/ml AHT and incubation over night at 30°C
  - The AHT will induce the I-*Sce*I on pWRG99 which selects for successful transformants
- Purify big colonies
- Verify clones by colony PCR, digestion of PCR products (if new restriction site was introduced) and final sequencing
- Curing of pWRG99 by restreaking and incubation at 37°C over night
- Test clones for carbenicillin sensitivity

**Supplementary References**

1. Gerlach RG, Jäckel D, Hölzer SU, Hensel M (2009) Rapid oligonucleotide-based recombineering of the chromosome of *Salmonella enterica*. Appl Environ Microbiol 75: 1575-1580.

2. Datsenko KA, Wanner BL (2000) One-step inactivation of chromosomal genes in *Escherichia coli* K-12 using PCR products. Proc Natl Acad Sci U S A 97: 6640-6645.

3. Maloy SR, Stewart VJ, Taylor RK (1996) Genetic Analysis of Pathogenic Bacteria: A Laboratory Manual. New York: Cold Spring Harbor Laboratory Press. 603 p.
